# Decreased expression of *lethal giant larvae* causes ovarian follicle cell outgrowth in the *Drosophila Scutoid* mutant

**DOI:** 10.1371/journal.pone.0188917

**Published:** 2017-12-20

**Authors:** Chen-Yuan Tseng, Hwei-Jan Hsu

**Affiliations:** Institute of Cellular and Organismic Biology, Academia Sinica, Taipei, Taiwan; Biocenter, Universität Würzburg, GERMANY

## Abstract

Snail, a zinc-finger transcription factor, controls the process of epithelial-mesenchymal transition, and ectopic expression of this protein may produce cells with stem cell properties. Because the effect of Snail expression in ovarian epithelial cells remains unclear, we generated *Drosophila* ovarian follicle stem cells (FSCs) with homozygous *Scutoid* (*Sco*) mutation. The *Sco* mutation is a reciprocal transposition that is known to induce ectopic Snail activity. We found that *Sco* mutant FSCs showed excess proliferation and high competitiveness for niche occupancy, and the descendants of this lineage formed outgrowths that failed to enter the endocycle. Surprisingly, such phenotypes were not rescued by suppressing Snail expression, but were completely restored by supplying Lethal giant larvae (Lgl). The *lgl* allele is a cell polarity gene that is often mutated in the genome. Importantly, *Sco* mutants survived in a complementation test with *lgl*. This result was probably obtained because the *Sco*-associated *lgl* allele appears to diminish, but not ablate *lgl* expression. While our data do not rule out the possibility that the *Sco* mutation disrupts a regulator of *lgl* transcription, our results strongly suggest that the phenotypes we found in *Sco* mutants are more closely associated with the *lgl* allele than ectopic Snail activity.

## Introduction

Epithelial-mesenchymal transition (EMT) is a highly conserved process in which immotile epithelial cells lose cell polarity and adhesion capability, becoming migratory mesenchymal cells [1]. Snail induces EMT by transcriptionally repressing E-cadherin [2, 3]. Recent data have shown that overexpression of Snail in tumor cell lines induces cell invasion, and cancer stem cell properties [4, 5]. We explored whether Snail dysregulation is sufficient to induce EMT, or a similar process, in non-cancerous epithelial cells, such as those derived from the *Drosophila* follicle cell lineage.

**Fig 1:**
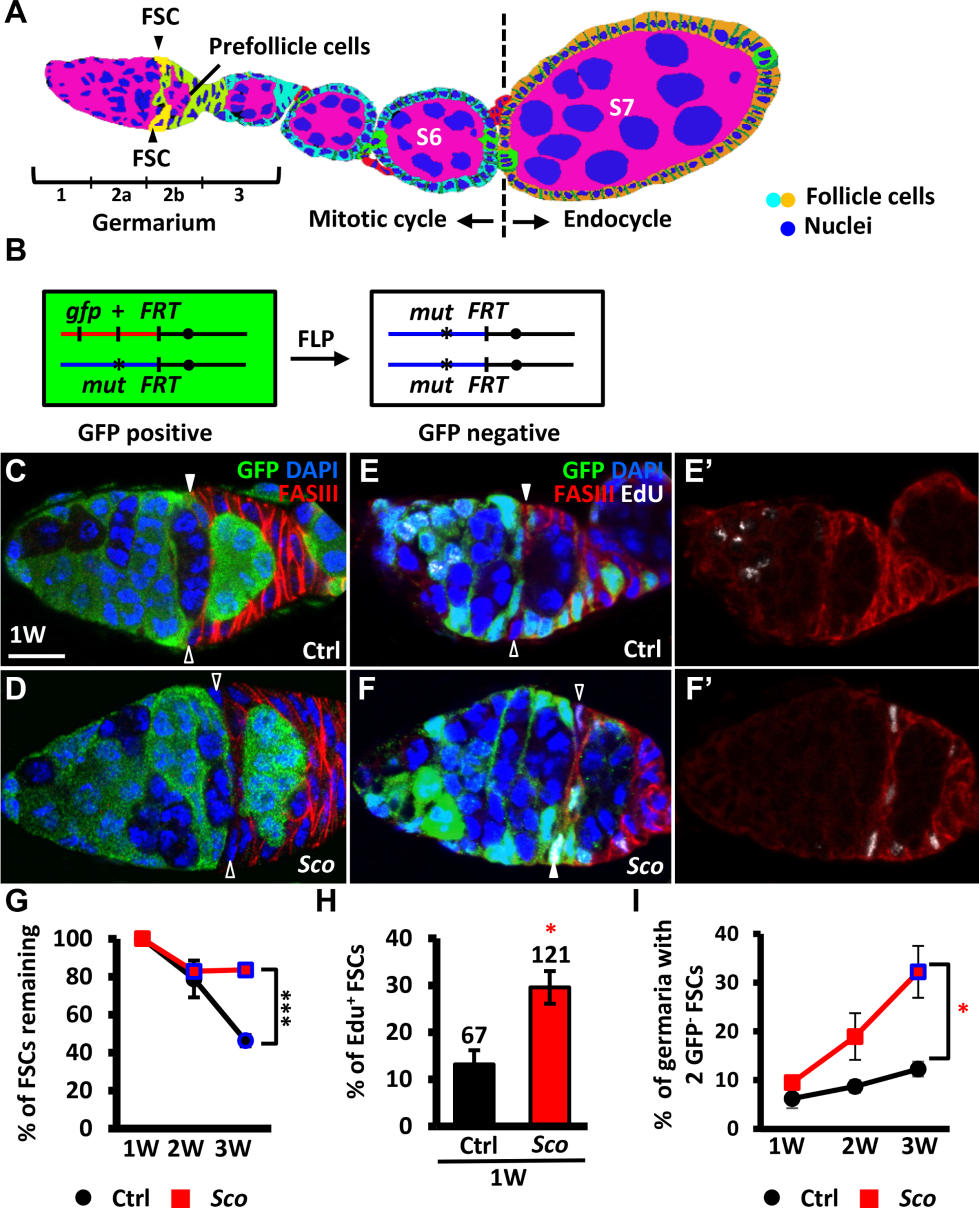
*Sco* FSCs exhibit extended lifespan, enhanced proliferation, and increased competitiveness for niche occupancy. (A) Schematic of the *Drosophila* ovariole. The anterior-most structure of the ovariole, the germarium, contains germ cells that are enveloped by prefollicle cells (light green), to form egg chambers. The prefollicle cells are derived from two FSCs (yellow), which are located at the 2a/2b boundary of the germarium. Follicle cells of egg chambers up to stage (S) 6 (light blue) undergo mitotic cycles, while follicle cells of egg chambers after stage 7 (orange) enter the endocycle. (B) Mitotic recombination was used to generate *Sco* FSCs. Females were generated, carrying a wild-type allele linked to a marker gene (GFP) *in trans* with the *Sco* allele. FLP-mediated recombination between *FRT* sites during mitotic division generated a homozygous *Sco* FSC that could be identified by the absence of GFP. (C-F) Control (Ctrl) (C and E) and *Sco* mosaic germaria (D and F) shown at one week (W) after clone induction (ACI): GFP (green, wild-type cells), FasIII (red, follicle cell lineages), DAPI (blue, DNA), and Edu (white in E and F, indicating proliferating cells). Solid and empty triangles indicate GFP-positive and GFP-negative FSCs, respectively. Scale bar, 10 μm. (G) Relative percentage (%) of germaria carrying GFP-negative follicle cell clones at 1, 2 and 3 weeks ACI. (H) Percentage of Edu-positive FSC clones in total FSC clones at one week ACI. The number of FSC clones analyzed is shown above the bar. (I) Percentage of germaria carrying two GFP-negative FSCs in control (black line) and *Sco* (red line) at 1, 2 and 3 weeks ACI. The blue squares in G and I indicate significant differences as compared to the initial time point. * *P*<0.05. *** *P*<0.001. Statistical analysis was carried out with student *t*-test. Data are shown as mean ± SEM. The genotype of C and E is *hs-flp*/+; *ubi-gfpFRT40A*/*FRT40A*, of D and F is *hs-flp/+; ubi-gfpFRT40A/ScoFRT40A*.

The *Drosophila* ovary is an excellent model by which to study the biology of epithelial cells [6]. Each ovary carries 15 to 20 ovarioles (Fig 1A), which are the functional units that continuously produce eggs [7]. The anterior-most structure of the ovariole, named the germarium, houses two or three germline stem cells (GSCs) at its tip. The immediate GSC progeny, called a cystoblast, divides four times to produce a 16-cell germline cyst. This germline cyst is then surrounded by prefollicle cells and buds off from the germarium to become an egg chamber, which passes through 14 different stages and finally develops into a mature egg. Prefollicle cells are derived from two follicle stem cells (FSCs) that are located on opposite sides of the junction between the 2a and 2b regions of the germarium [8, 9]. Shortly after surrounding the germline cyst, prefollicle cells differentiate into stalk cells, polar cells, and follicle cells. Stalk cells link egg chambers, while two polar cells located at the anterior and posterior poles of the egg chamber function to control follicle cell fate and anterior-posterior axis determination. The follicle cells form a polarized epithelium around each egg chamber [8]. Before stage 6, follicle cells undergo a mitotic cycle that includes the complete set of G1, S, G2 and M phases [10], whereas around the beginning of stage 7, the follicle cells enter an endocycle, which includes only the G and S phases [11].

In our previous study, overexpression of *snail* in the follicle cell lineage by the UAS/GAL4 system only resulted in increased proliferation of FSCs and mild outgrowth of stalk cells [12]. However, the increased activity of Snail in this model may not be sufficient to drive EMT in follicle cells, as E-cadherin expression was only slightly reduced [12]. The dominant *Scutoid* (*Sco*) mutation is a reciprocal transposition of two small regions and results in ectopic Snail activity (plane A and B in S1 Fig) [13]. In this study, we generated FSCs that were homozygous for the *Sco* mutation and traced them and their progeny. We found that these *Sco* mutant FSCs were hyperproliferative, resulting in outgrowth and increased niche occupancy. *Sco* mutant follicle cells also did not enter the endocycle and lost cell polarity. However, none of the phenotypes were rescued by suppressing Snail activity. To our surprise, follicle cell defects could be completely rescued by exogenous expression of *lethal giant larvae* (*lgl*), a cell polarity gene that is often spontaneously mutated. Our results indicate that ectopic Snail activity is not responsible for the observed outgrowth of *Sco* mutant follicle cells, and does not drive EMT in ovarian epithelial cells. However, *lgl* may be a second-site allele that is associated with the *Sco* mutant and substantially contributes to the observed phenotypes. Importantly, this allele could not be identified by simple complementation test.

## Results and discussion

### *Sco* mutant FSCs exhibit increased proliferation and extended lifespan

To determine whether forcing Snail activity induces EMT in the follicle cell lineage, we used a FLP-mediated recombination technique to generate GSCs homozygous for *Sco* (Fig 1B). The *Sco* mutant cells could be recognized by the absence of GFP (Fig 1C–1F) and their localization in the tissue. FSCs lack specific molecular markers, and therefore cannot be unambiguously identified. However, these cells can be indirectly recognized based on their location at the border of germarial regions 2a and 2b, where FasIII, a marker for prefollicle cells, is weakly expressed. FSCs are the cells immediately anterior to the FasIII-positive prefollicle cells [9, 14]. In addition, prefollicle cells eventually differentiate and leave the germarium after three to four days [14], while FSCs are retained. Therefore, beginning at one week after clone induction (ACI), the presence of prefollicle cell clones (GFP-negative) can be an indicator of the existence of GFP-negative FSCs.

We first examined the maintenance of *Sco* mutant FSCs by assessing the percentage of germaria carrying *Sco* mutant prefollicle cell clones over time (Fig 1G and S1 Table). At three weeks ACI, 46% of *FRT40* control germaria (*n* = 205) retained at least one wild-type control FSC that was generated during the first week (Fig 1G). This result indicates that up to 54% of FSCs had undergone natural turnover in the controls, which is consistent with an earlier report that the half-life of FSCs is two to three weeks [15]. However, 83% of *Sco* mutant FSCs remained in *Sco* mutant mosaic germaria (*n* = 163) at three weeks ACI, suggesting that *Sco* mutant FSCs have a prolonged lifespan. We also examined the division rate of *Sco* mutant FSCs using a mitotic marker, phospho-Histone3 (PH3), to label cells in M phase, and a DNA replication marker, EdU incorporation, to show cells in S phase (Fig 1E and 1F). We did not observe any PH3 positive FSCs in control mock or mutant mosaic germaria at one week ACI (control *n* = 183, mutant *n* = 252; S1 Table), probably due to the short duration of mitosis. These results are consistent with those of a previous report [16]. However, at the same time-point, the frequency of EdU positive *Sco* mutant FSCs in *Sco* mutant mosaic germaria was 2.3-fold greater than that of control FSCs in mock mosaic germaria (Fig 1H; 13.1% for control, *n* = 67 vs. 29.5% for *Sco* mutant, *n* = 121), indicating that homozygous *Sco* mutant FSCs underwent rapid division.

Interestingly, we also observed that the proportion of *Sco* mutant mosaic germaria, in which all FSCs were mutant (i.e. two GFP-negative FSCs), increased from 10% (*n* = 193 germaria) at one week to 32% (*n* = 163 germaria) by three weeks ACI (Fig 1I and S1 Table). In *FRT40A* mock mosaic germaria, a much smaller increase was observed (one week ACI: 6.2%, *n* = 176 germaria vs. three week ACI: 13.3%, *n* = 155 germaria). This small increase was probably due to natural loss, which arose from replacement of opposite GFP-negative FSCs with GFP-positive FSCs from the same germarium (Fig 1I). These results indicate that *Sco* mutant FSCs are more competitive for niche occupancy and tend to replace wild-type FSCs. Furthermore, it is possible that their progeny exhibit increased chance of migrating across the germline to compete in the FSC niche [9].

### *Sco* mutant follicle cells form outgrowths that fail to enter the endocycle

**Fig 2:**
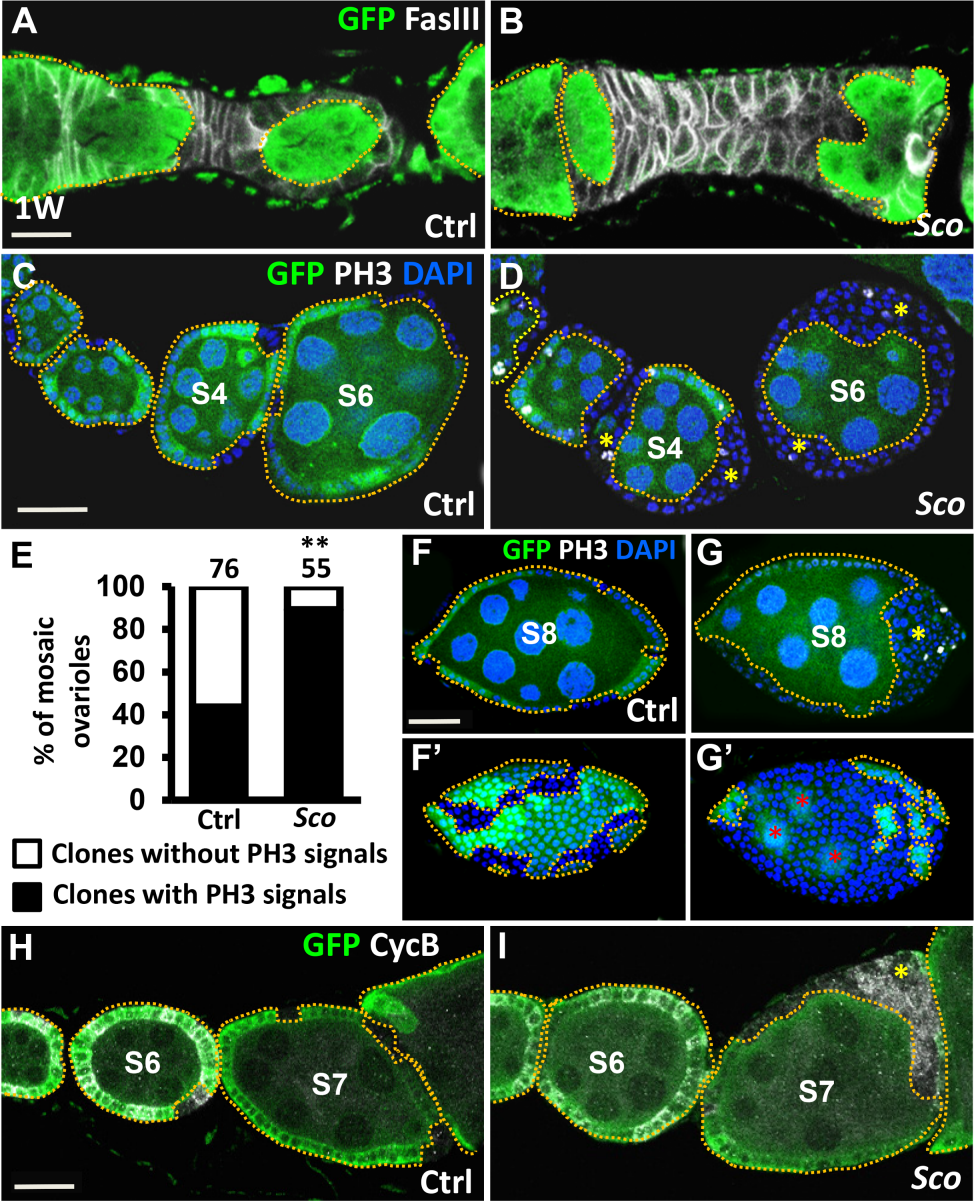
*Sco* follicle cells are hyperproliferative and do not enter the endocycle. Control (Ctrl) (A, C, F and H) and *Sco* mosaic ovarioles (B, D, G and I) at one week (1W) after clone induction (ACI) are labeled with GFP (green, wild-type cells) and FasIII (gray, membranes of follicle cell lineages) in A and B, phospho-Histone 3 (PH3, gray, mitotic marker) and DAPI (blue, DNA) in C, D, F and G, and Cyclin B (CycB, gray, G2/M phase marker) in H and I. Wild-type cells are outlined by yellow dashed lines. The scale bar in A is 10 μm, and scale bars in C, F and H are 20 μm. (A and B) *Sco* mosaic ovarioles contain stalk cell overgrowths that are completely composed of excessive numbers of irregularly-shaped *Sco* cells, as compared to the control. (C and D) *Sco* follicle cells formed multiple layers in stage (S) 4 and 6 egg chambers. (E) Percentage (%) of mosaic ovarioles exhibiting PH3 signal in GFP-negative follicle cell clones. The number of ovarioles analyzed is shown above each bar. (F and G) *Sco* follicle cells of the stage 8 egg chamber formed multiple layers, and continued to undergo mitosis. F’ and G’ show the outermost layers of egg chambers. Asterisks indicate nurse cells. (H and I) *Sco* follicle cells of the stage 7 egg chamber retained greater CycB signals than the controls. Wild-type cells are outlined by yellow dashed lines. Asterisks indicate anterior or posterior poles of egg chambers. The genotype of the controls in A, C, E, F and H is *hs-flp*/+; *ubi-gfpFRT40A*/ *FRT40A*, and of the *Sco* mosaic mutant in B, D, E, G and I is *hs-flp/+; ubi-gfpFRT40A/ScoFRT40A*.

We noticed that progeny derived from *Sco* mutant FSCs, including stalk cell precursors (generated from prefollicle cell intermediates) and follicle cells (which cover egg chambers), formed outgrowths. Compared to the control, the number of stalk cell precursors in *Sco* mutants was dramatically increased (Fig 2A and 2B). In addition, while normal follicle cells in the control mosaic ovariole undergo mitotic division from the germarium to stage 6 egg chambers, forming a monolayer that covers germ cells (Fig 2C), *Sco* mutant follicle cells formed multiple layers at the anterior and posterior poles of stage 4 and 6 egg chambers, carrying wild-type germ cells (Fig 2D). A total of 44% of control mock mosaic ovaries (*n* = 76) carried follicle cell clones with PH3 signal. This was compared with 89% of *Sco* mutant mosaic ovaries (*n* = 55) carrying *Sco* mutant follicle cells with PH3 signals (*P* < 0.01; Fig 2E), indicating a high division rate for the *Sco* mutant cells. We did not observe obvious phenotypes for egg chambers with *Sco* mutant germ cells that were surrounded by normal follicle cells (plane C-F in S1 Fig), suggesting a specific effect of *Sco* on follicle cells.

After stage 6, follicle cells transition from the mitotic cell cycle to the endocycle [10]. As such, stage 8 control mock mosaic egg chambers do not carry PH3-positive follicle cells (Fig 2F). However, *Sco* mutant follicle cell clones within the stage 8 egg chamber still expressed PH3 (Fig 2G). Moreover, clonal size was enlarged in the mutants (Fig 2F’ and 2G’), suggesting that *Sco* mutant cells continuously proliferate to edge out wild-type cells. Similarly, Cyclin B, a G2/M phase marker [17], is normally only expressed in follicle cells until stage 6 (Fig 2H, *n* = 11). However, Cyclin B expression was detected in *Sco* mutant follicle cells of all the stage 7 egg chamber (Fig 2I, *n* = 23), indicating that *Sco* mutant follicle cells fail to switch from a mitotic cycle to an endocycle.

**Fig 3:**
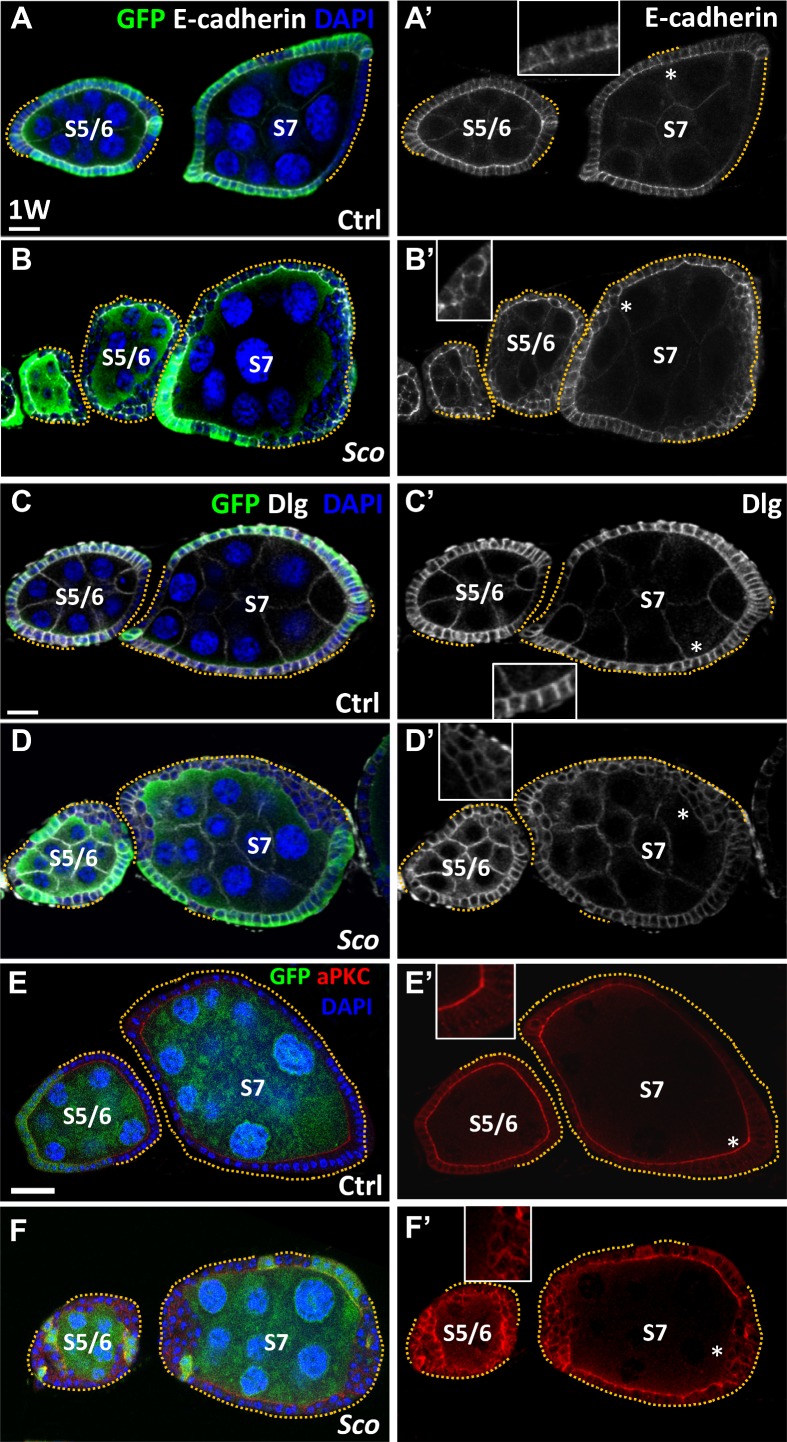
*Sco* follicle cells lose cell polarity and are delaminated. (A-F) One-week (w)-old control (ctrl) (A, C and E) and *Sco* mosaic (B, D and F) stage (S) 6 and 7 egg chambers: GFP (green, wild-type cells) and E-cadherin (gray, E-cad) in A and B; Disc large (gray, Dlg) in C and D; aPKC (E and F); DAPI (blue, DNA). A’ and B’ show E-cad channel only; C’ and D’ show Dlg channel only; E’ and F’ show aPKC only. Dashed lines indicate follicle cell clones. Inserts are enlarged images from the area indicated by asterisks. Scale bar, 20 μm. The genotype of A, C and E is *hs-flp*/+; *ubigfp FRT40A*/ *FRT40A*, of B, D and F is *hs-flp/+; ubi-gfpFRT40A/ScoFRT40A*.

**Fig 4:**
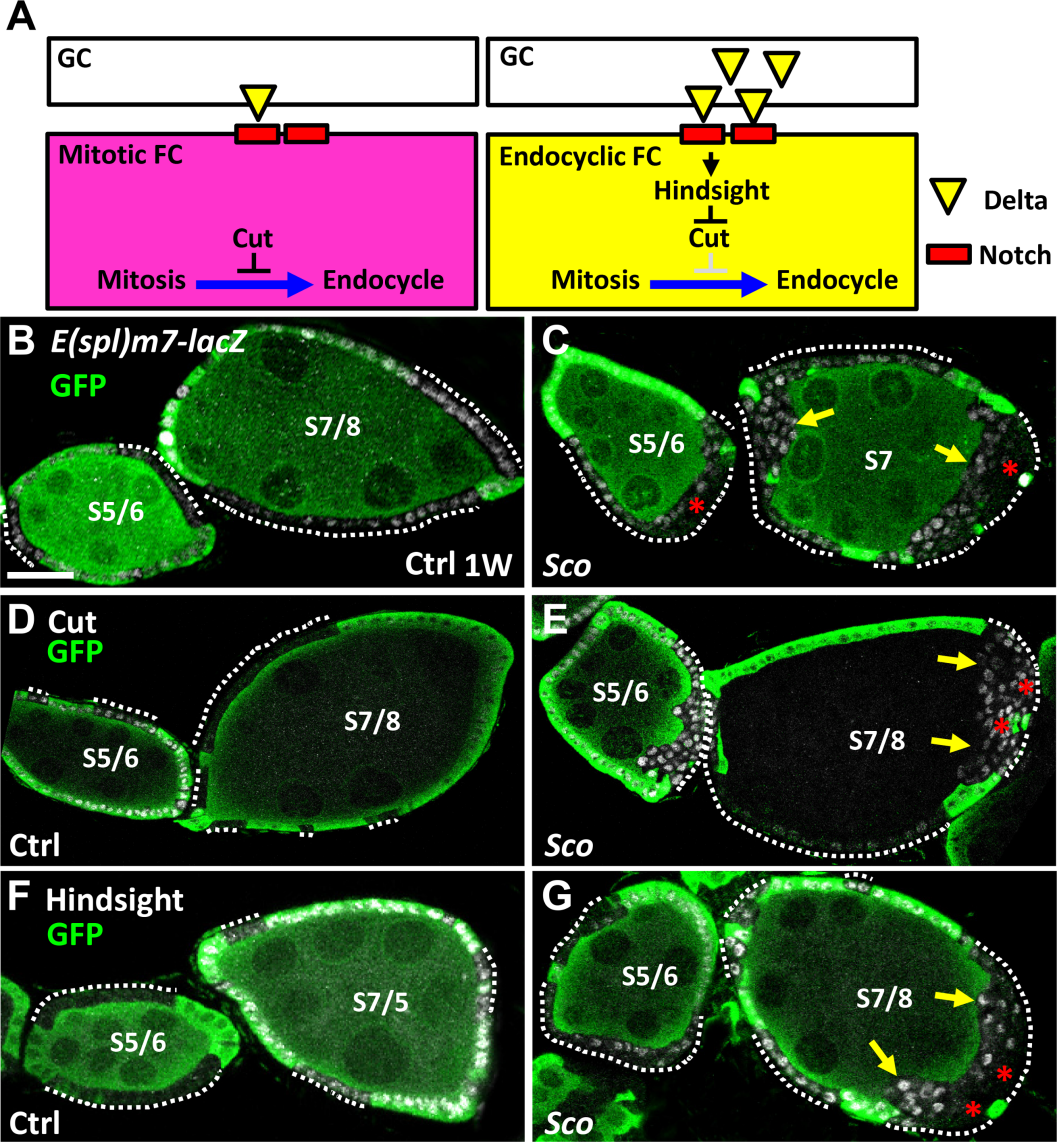
Notch signaling is disturbed in the outer layers of *Sco* follicle cells. (A) Notch signaling is required for the transition of follicle cells from the mitotic phase to endocycle phase [10]. In mitotic follicle cells (up to stage 6), Notch signaling activity is low because germ cells produce low amounts of Delta (a Notch ligand, shown as yellow triangles). In addition, Cut is expressed in follicle cells to suppress the mitosis-endocytosis transition. After stage 7, Notch signaling is activated to promote Hindsight expression, which suppresses Cut expression and thereby permits the mitosis-endocytosis transition. Red squares indicate Notch receptors. (B-G) One-week (w)-old control (ctrl) (B, D and F) and *Sco* mutant mosaic egg chambers (C, E and G) at stages (S) 6 and 7: GFP (green, wild-type cells), *Esplm7*-*lacZ* (gray, a Notch signaling reporter) in B and C, Cut (gray) in D and E, Hindsight (gray) in F and G. Expression of *E(spl)m7-lacZ* is decreased in ectopic layers of *Sco* mutant follicle cells located far from the germline at the anterior and posterior poles. In the control, Cut is mainly expressed in stage 6 follicle cells and is downregulated in stage 7 follicle cells. However, Cut expression is weaker in the inner layer as compared to the outer layer of *Sco* mutant follicle cells. In contrast, Hindsight expression is stronger in the inner layer as compared to the outer layer of *Sco* follicle cells at stage 7, indicating a non-cell autonomous effect of *Sco* on the mitosis-endocycle transition. Arrows show the boundary between germ cells and follicle cells. Asterisks indicate outer layers of ectopic follicle cells. Scale bar, 20 μm. The genotype of B, D and F is *hs-flp*/+; *ubi-gfpFRT40A*/*FRT40A*, of C, E and G is *hs-flp/+; ubi-gfpFRT40A/ScoFRT40A*.

Interestingly, all *Sco* mutant follicle cells lost cell polarity, as evidenced by dysregulated E-cadherin (E-cad; Fig 3A, *n* = 30, and B, *n* = 27), Disc large (Dlg; Fig 3C, *n* = 11, and D, *n* = 19) and Atypical protein kinase C (aPKC; Fig 3E, *n* = 5, and F, *n* = 7) expression. The loss of all of these factors may also contribute to the failure of the mitotic-endocycle transition [18, 19]. E-cad and Dlg are polarity genes that are mainly enriched in apical-lateral and lateral domains of follicular cells, respectively [19–21]. However, expression of E-cad and Dig was not restricted to specific subcellular domains in *Sco* mutant follicle cells (Fig 3A–3D). aPKC is mainly expressed in the apical domain of follicle cells [19], but similar to the other two markers, it was not restricted to a specific domain of *Sco* mutant follicle cells (Fig 3E and 3F). In addition, Notch signaling is a key factor in the control of the mitotic-endocycle transition [10, 22, 23]. In mitotic follicle cells of stage 1 to 6 egg chambers, Cut is expressed and suppresses early entry into the endocycle. After stage 6, follicle cells receive Delta from germ cells and activate Notch signaling, which upregulates expression of Hindsight to suppress Cut expression and initiate entry into the endocycle (Fig 4A). We found that Notch signaling, as monitored by the *E(spl)m7-lacZ* Notch reporter [24], was not activated in the outer layer of all *Sco* mutant follicle cells (Fig 4B, *n* > 20, an C, *n* > 20). Consequently, Cut expression was retained (Fig 4D, *n* > 20, and E, *n* > 20), and Highlight expression was absent from the outer layer of all *Sco* mutant follicle cells of stage 7 egg chambers (Fig 4F, *n* > 20 and G, *n* > 20). To summarize, we observed that *Sco* mutant FSCs lost cell polarity, formed outgrowths and failed to undergo the mitosis-endocycle transition. Together, these observations strongly suggest that EMT, or an EMT-like process, occurs within *Sco* mutant cells.

### Snail and Noc do not account for the multiple-layered phenotype of *Sco* mutant follicle cells

**Fig 5:**
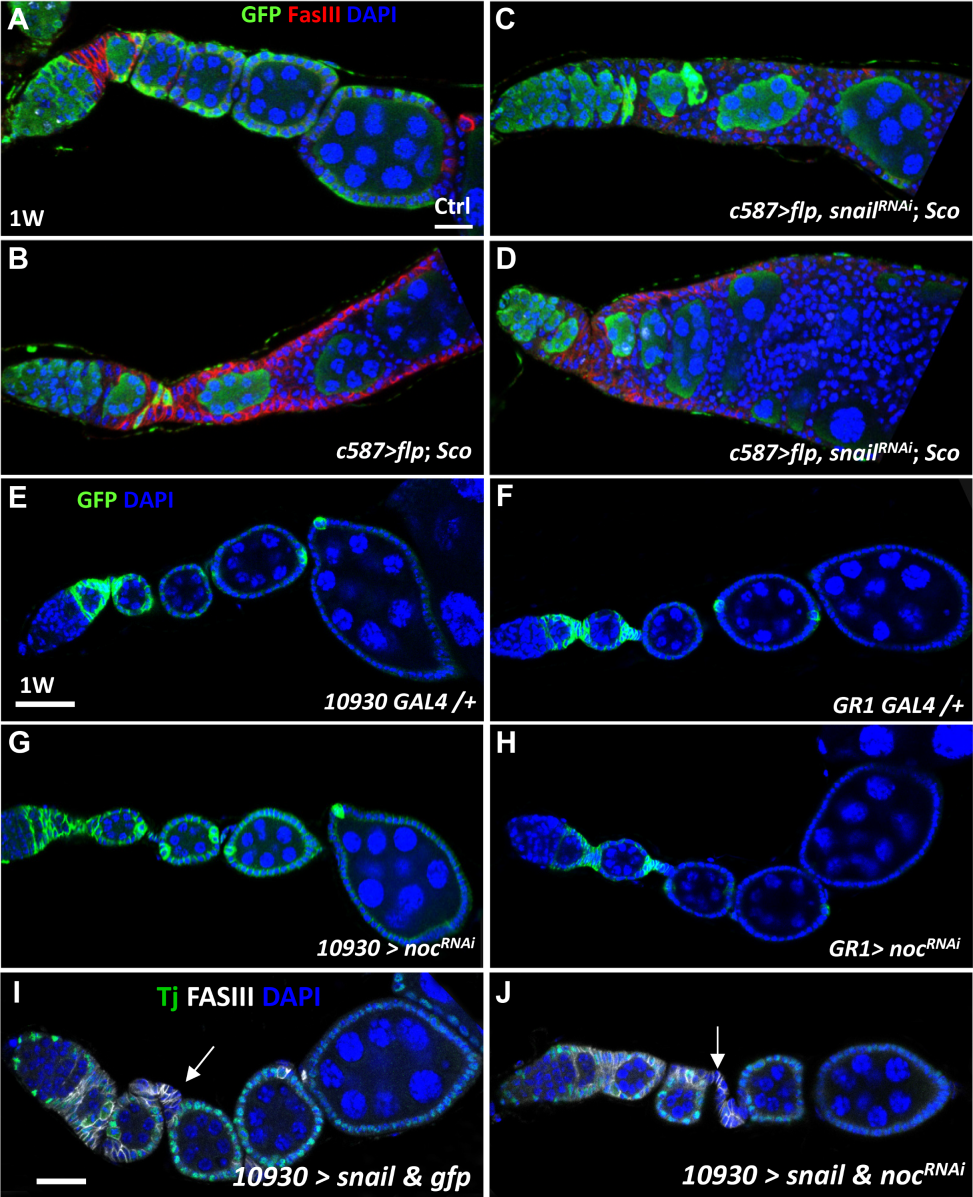
Snail and Noc do not account for the multiple-layered phenotype of *Sco* follicle cells. (A-D) One-week-old control (ctrl) mosaic (A), *Sco* mosaic (B) and *Sco* mosaic ovarioles with *snail* knockdown (C and D). (E-J) One-week (W)-old control (ctrl) (E and F), *10930>noc*^*RNAi*^ (G), *GR1> noc*^*RNAi*^ (H), *10930>snail & gfp* (I), and *10930>snail & noc*^*RNAi*^ ovarioles (J): FasIII (green, follicle cell lineages), Traffic Jam (Tj) (gray, follicle cells) in I and J, and DAPI (blue, DNA). Arrows in I and J indicated overexpression of Snail increase cell number in the stalk that connects two egg chambers. The scale bar is 20 μm. The genotype of A is *c587-GAL4/UAS-flp; ubi-gfpFRT40A/FRT40A*, of B, C, and D is *c587-GAL4/UAS-flp*: *ScoFRT40A/ubi-gfpFRT40A*.

The *Sco* mutant chromosome is induced by the transposition of two DNA fragments, causing fusion of the *snail* and *no ocelli* (*noc*, encoding a zinc finger protein belonged to the NET family) genes [13]. It has been shown that the phenotype of mechano-bristle loss in the thorax and eyes of *Sco* heterozygous flies can be rescued by decreasing expression of *snail* or increasing expression of *noc* [13], indicating that Snail is ectopically expressed while Noc expression is reduced in *Sco* mutants. We therefore hypothesized that these two genes may be responsible for the phenotype we observed in the *Sco* mutant follicle cell lineage. To test this possibility, we knocked down *snail* in FSCs and their progeny that were homozygous for *Sco* mutations using the FLP/FRT system under the control of *c587-GAL4*, which is expressed in the follicle lineage (S2 Fig). However, knockdown of *snail* did not prevent *Sco* mutant follicle cells from forming multiple layers (Fig 5A to 5D; control: *n* = 10, *Sco*: *n* = 10, and *Sco* with *Snail*^*RNAi*^: *n* = 10). We then attempted to phenocopy the *Sco* mutation by knocking down *noc* expression in the follicle cell lineage using *109–30 GAL4* (Fig 5E and 5G), which is expressed in FSCs and early follicle cells, or *GR1 GAL4* (Fig 5F and 5H), which is expressed from stage 3 to stage 10 follicle cells [25, 26]. However, neither the elimination of *noc* (Fig 5G, *n* > 20, and H, *n* > 20), nor exogenous expression of *snail* with decreased *noc* expression could cause the formation of multiple layers of follicle cells (Fig 5I, *n* > 20, and J, *n* > 20). Notably, overexpression of *snail* did induce increased cells numbers in the stalk that connects two egg chambers in approximately 30% of the ovarioles (arrows in Fig 5I, *n* > 20, and G, *n* > 20). Therefore, the phenotype caused by the *Sco* chromosome is complex and not fundamentally reliant on *snail* and *noc*, suggesting that the EMT-like phenotype may require the disruption of a tumor suppressor gene.

### Lethal giant larvae controls follicle cell homeostasis in the *Sco* stock

**Fig 6:**
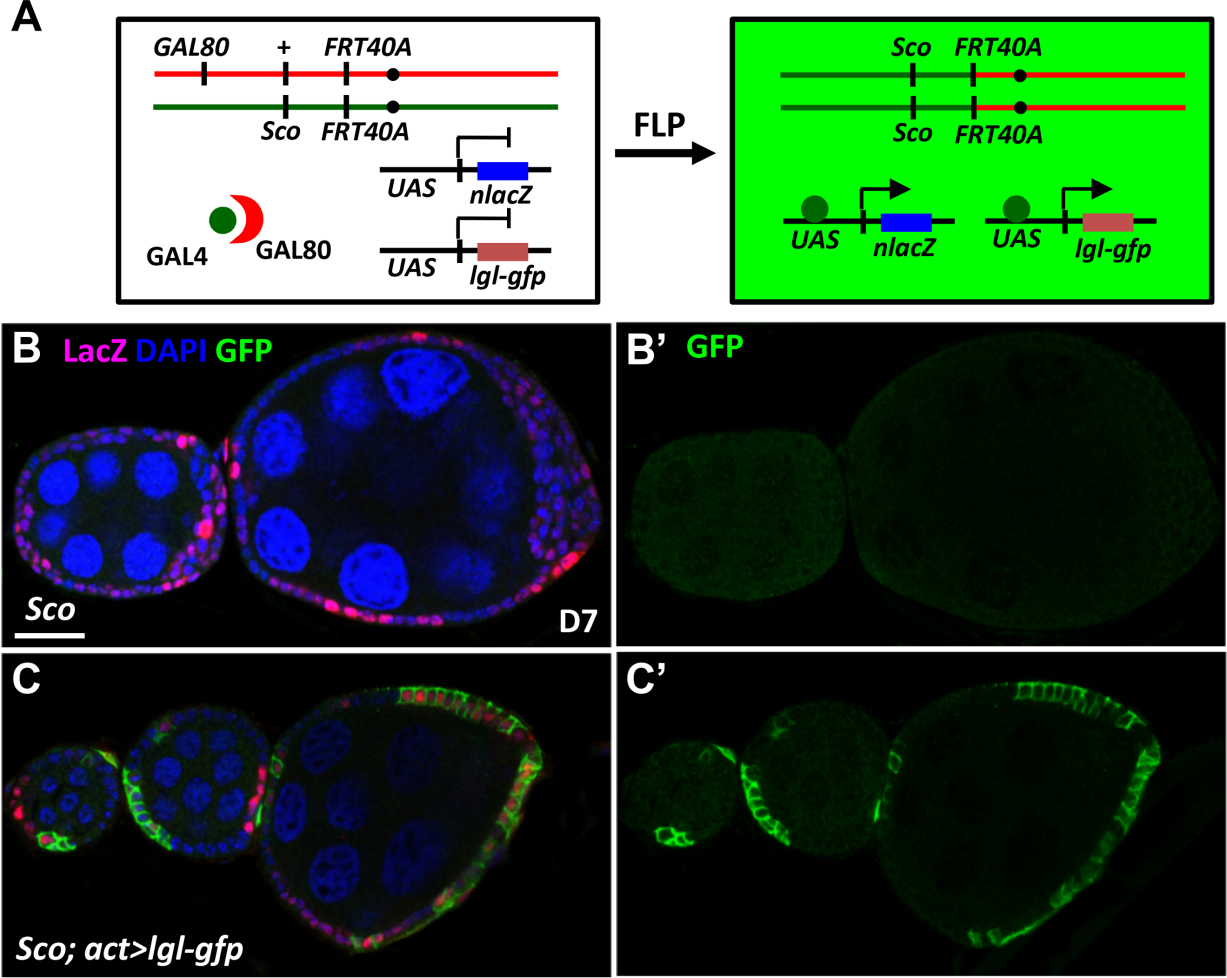
Exogenous supplement of *lgl* prevents *Sco* follicle cells forming multiple layers. (A) Mosaic Analysis with Repressible Cell Marker (MARCM) was used to generate *Sco* homozygous mutant cells expressing *lgl-gfp*. Females carried wild-type alleles linked to *tubulin promoter-GAL80* (a GAL4 suppressor) *in trans* with the *Sco* mutant allele on the second chromosome, *actin promoter-GAl4*, *UAS-nuclear (n) LacZ* and *UAS-lgl-gfp* on the third chromosome. FLP-mediated recombination between two FRT sites during mitotic division generated *Sco* homozygous mutant cells lacking GAL80, allowing *UAS*-trangenes to be expressed by GAL4 driven by an *actin* promoter. (B-C) Mosaic egg chambers in one-week (W)-old *Sco* mutant (B), and *Sco* mutant with *lgl* overexpression: LacZ (green, mutant cells) and DAPI (blue, DNA). Dashed lines mark follicle cell clones. The scale bar is 20 μm. The genotype of B is *hs-flp*/+; *tub-Gal80FRT40A*/ *ScoFRT40A*; *act-GAL4UAS-nlacZ/+*, and of C is *hs-flp*/+; *tub-Gal80FRT40A*/ *ScoFRT40A*; *act-GAL4UAS-nlacZ/UAS-lgl-gfp*.

The *Sco* allele is located on the left arm of Chromosome II, which also contains *lgl*. This gene is frequently found to be spontaneously mutated in *Drosophila* stocks [27], due to its location as the second protein-coding gene downstream of the sub-telomeric region of chromosome 2L [28]. Mutations in *lgl* have been shown to produce long stayed FSCs [29], multiple-layered follicle cells [30] and failure to enter the endocycle [10, 18]. To determine if the *Sco* stock we used contains an *lgl* mutation, which could explain the previously described phenotypes, we first performed a complementation test. We crossed *Sco FRT40A/CyO* flies with flies heterozygous for *lgl*^*4*^, a null allele [31], balanced by *CyO*. We then examined the generation of *ScoFRT40A/lgl*^*4*^ flies. Because *lgl*^*4*^ is homozygous lethal, we should not obtain *Sco FRT40A/lgl*^*4*^ flies if the chromosome carries both *Sco FRT40A* and a strong *lgl* mutation. The ratio of three genotypes we obtained from the progeny were as follows: *Sco FRT40A /CyO*, 2 ± 4%; *lgl*^*4*^*/CyO*, 57 ± 2%; and *Sco FRT40A /lgl*^*4*^, 41 ± 6% (333 flies were analyzed for each test). Since we clearly observed that *Sco FRT40A /lgl*^*4*^ flies were frequently produced, the result suggested that the *Sco* phenotypes we observed in FSCs and their progeny may not be due to the mutation of *lgl*. However, it remained possible that this test may not detect a weak *lgl* allele. To test this, we generated the *Sco* mutant follicle cell lineage expressing *lgl-gfp*, using the Mosaic Analysis with Repressible Marker (MARCM) technique (Fig 6A). Surprisingly, the multiple-layered phenotype of *Sco* mutant follicle cell clones was completely rescued by exogenous expression of Lgl (Fig 6B, *n* = 13, and C, *n* = 24). In addition, we did not observe an increased number of stalk precursors in *Sco* mutant plus Lgl overexpression mosaic ovarioles, indicating that Lgl supplementation also rescues the *Sco* mutant stalk cell phenotypes. Consistent with this rescue effect, expression of *lgl* in the ovaries carrying *Sco* mutant follicle cell lineages (FPKM value: 31) was less than half of that in the control ovaries (FPKM value: 69, *P*<0.0001), as analyzed by RNAseq. These results strongly suggest that a second allele mutation in *lgl* accounts for the phenotypes that we observed in the *Sco* mutant follicle cell lineage. However, we cannot rule out the possibility that the *Sco* mutation may directly or indirectly disrupt some regulator of *lgl* expression.

Following our observation that *lgl* supplementation can rescue the phenotype in *Sco* mutant FSC lineage cells, we carefully compared the phenotype of *lgl* mutant FSCs and their lineage that was reported by previous studies [18, 29] and the *Sco* mutants. This comparison revealed two major differences between *lgl* and *Sco* mutant FSCs and follicle cells. First, although both *lgl* and *Sco* mutant FSCs exhibit prolonged maintenance and increased competition for niche occupancy, the *lgl* mutation does not affect FSC proliferation, while the *Sco* mutation results in a two-fold increase in FSC proliferation. Second, although both *lgl* and *Sco* mutant follicle cells form multiple layers in egg chambers, 61% of *lgl* mutant mosaic ovarioles carry fused egg chambers that are not observed in *Sco* mutant mosaic ovarioles. We have previously demonstrated that Snail promotes FSC proliferation [12], suggesting that *Sco* mutant FSCs may exhibit a combined phenotype that results from high Snail and low Lgl activity.

It has been previously reported that a high frequency of *lgl* alleles exist in wild populations of *Drosophila melanogaster*, as well as the Bloomington second chromosome deficiency kit and the University of California at Los Angeles Bruinfly *FRT40A*-lethal P collection [27]. Here, we also report that a weak *lgl* mutant allele may be associated with the Bloomington *Sco* stock. These results emphasize the need to routinely test second chromosome stocks for second-site alleles of *lgl*. Furthermore, it is important to note that simple complementation tests may not be enough to identify mutant *lgl* alleles.

## Materials and methods

### Fly stocks and culture

Flies were cultured at 22–25°C on standard medium, unless otherwise indicated. *w*^*1118*^ was used as wild-type controls. *Sco* is a chromosome rearrangement mutant generated by X-ray-induced mutagenesis; *ScoFRT40A* was obtained from the Bloomington fly stock center (B 5759) [13, 32]. *UAS-RNAi* lines against *snail* (VDRC 50003) and *noc* (VDRC 108422) were obtained from the Vienna *Drosophila* RNAi Center; their efficiencies were described previously or tested here [33, 34]. *c587-GAL4*, *10930-GAL4*, *GR1-GAL4*, and *UAS-lgl-gfp* have been previously described [24–26, 35, 36]. *E(spl)m7-lacZ* was used to monitor Notch signaling [33]. Food was changed daily until dissection. Other genetic elements are described in Flybase (http://flybase.bio.indiana.edu).

### Genetic mosaic analysis

Mosaic clones were generated by Flipase (FLP)/FLP recognition target (*FRT*)-mediated mitotic recombination [37]. For conventional mosaic analysis, females of genotype *hs-flp/+; ubiGFPFRT40A/ScoFRT40A* and *hs-flp/+; ubiGFPFRT40A/FRT40A*, *c587*,*UAS-flp/+; ubiGFPFRT40A/ScoFRT40*,*c587*,*UAS-flp/snail*^*RNAi*^*;ubiGFPFRT40A/ScoFRT40A* were generated using standard crosses. For mosaic analysis with repressible marker (MARCM) [38], females of genotype *hs-flp/+; tubGAL80FRT40A/ScoFRT40A; actFRT-CD2-FRT-GAL4 UAS-nlacZ/ +*, and *hs-flp/ +; tubGAL80FRT40A/ScoFRT40A; actFRT-CD2-FRT-GAL4 UAS-nlacZ/ UAS-lgl-gfp* were generated. To generate conventional FSC clones, two-day-old female flies were subjected to heat shock at 37°C for 1 h, twice a day for three days. For MARCM experiments, two-day-old female flies were heat shocked at 37°C for 30 min. After heat shock, females were raised at 25°C and received fresh food daily until dissection. Homozygous mutant cells were recognized by the absence of GFP in conventional mosaic analysis, but identified by the presence of LacZ in MARCM. We were unable to directly identify FSCs in MARCM experiments, due to the weak expression of LacZ in FSCs.

### RNA sequencing analysis

Thirty pairs of ovaries were collected and dissected from 1-week old female flies that were cultured at 25°C. The genotypes of the flies were *c587-GAL4*, *UAS-flp/+; ubi-gfp FRT40A/FRT40* or *c587-GAL4*,*UAS-flp/+;ubi-gfp FRT40A/ScoFRT40A*. Total RNA were extracted by Trizol reagent (Invitrogen, USA) according to the instructions. RNA was quantified at by absorbance at 260 nm using a ND-1000 spectrophotometer (Nanodrop Techonology, USA) and quality was assessed using a Bioanalyzer 2100 (Agilent Technology, USA) with a RNA 6000 labchip kit (Agilent Technologies, USA). All RNAseq procedures were carried out according to the manufacturer’s protocol from Illumina. Library construction for all samples was accomplished with Agilent’s SureSelect Strand Specific RNA library Preparation Kit for 75SE (Single-End or Paired-End) sequencing on Solexa platform. The sequence was directly determined using sequencing-by-synthesis technology with a TruSeq SBS kit. Raw sequences were obtained from the Illumina Pipeline software bcl2fastq v2.0 and expected to generate 12.5M (million reads) per sample. The sequences were then filtered to obtain qualified reads. Trimmomatic software was implemented to trim or remove the reads according to the quality score. The gene expression level was calculated as FPKM (Fragment Per Kilobase of transcript per Million mapped reads). For differential expression analysis, CummeRbund was used to perform statistical analysis of gene expression profiles. The reference gene annotations were retrieved from Flybase. Data was deposited in the NCBI GEO under the accession number GSE43506.

### Immunostaining and fluorescence microscopy

Immunostaining and EdU incoporation in ovarian tissue was performed as previously described [12, 33]. The following primary antibodies were used: mouse anti-Fasciclin III (FasIII) (Developmental Studies Hybridoma Bank, DSHB, 1:50), Mouse anti-discs large (Dlg) (DSHB, 1:50), Mouse anti-cut (DSHB, 1:50), Mouse anti-hindsight (Hnt) (DSHB, 1:50), Rabbit anti-Phospho H3 (Millipore, 1:500), Rabbit anti-GFP (Torrey Pines, 1:1000), Mouse anti-β-gal (Promega, 1:500). The following secondary antibodies were used: AlexFluro 488-, 563-, and 633-conjugated goat species-specific secondary antibody (Molecular Probes, 1:1000). EdU incorporation was performed with the Click-iT Edu imaging kit (Invitrogen). Samples were stained with 0.5 μg/ml DAPI (Sigma) and mounted in 80% glycerol with 20 μg/ml N-propyl gallate (Sigma), and analyzed using a Zeiss LSM 710 confocal microscope.

Egg chamber stages were characterized as previously described [39, 40]. The stage 1 egg chamber resides in region 3 of the germarium; stages 2–7 are characterized by polyploidization of the nurse cells and mitotic division of the follicle cells, as well as increases in the size of the egg chamber. Stage 7 egg chambers are obviously elongated in shape. At the molecular level, stage 6 and 7 are distinguished by the expression of mitotic markers, Notch reporter, Cut and Hnt. Mitotic markers and Cut are expressed in stage 6, but diminished in stage 7; Notch reporter and Hnt start to be expressed in stage 7. The oocyte begins to accumulate yolk at stage 8. At stage 9, most of the follicle cells change from their original cuboidal shape to a columnar shape and are located at the posterior half of the egg chamber.

## Supporting information

S1 Fig *Sco* mutant flies exhibit a lack of bristles, due to ectopic Snail activity. (A) *Sco/+* flies lack anterior and posterior scutellar bristles. (B) Knockdown of *snail* using a *UAS-RNAi* line driven by *act-GAL4* restores apical and basal scutellar bristles in *Sco/+* flies. Asterisks indicate scutellum. (C-F) One-week-old stage 7 control (C and D) and Sco mutant mosaic egg chambers (E and F) with GFP (green, wild-type cells), FasIII (red, follicle cell lineage), and DAPI (blue, DNA). Dashed lines outline clones; scale bar, 20 μum. The genotype of flies in A are *Sco FRT40A/CyO*, B includes *snail*^*RNAi*^*/+*; *Sco FRT40A/+*; *act-GAL4/+*, C and D include *hs-flp*/+; *ubigfp FRT40A*/*FRT40A*, and E and F show *hs-flp/+; ubi = gfp FRT40A/ScoFRT40A*.
(TIF)

S2 Fig *c587-GAL4* is expressed in escort cell, FSCs and follicle cells of the early egg chamber. (A) 7-day-old *c587>gfp* germarium labeled with 1B1 (gray, fusomes and follicle cell membranes) and GFP (green). (A’) the image shown the GFP channel only. Scale bar, 20 μm.
(TIF)

S1 Table *Sco* mutant FSCs divide faster and persist longer than control FSCs. (DOCX)
