# Supplementary material for: Decreased expression of lethal giant larvae causes ovarian follicle cell outgrowth in the Drosophila Scutoid mutant
Source: PLoS One. 2017 Dec 20;12(12):e0188917. doi: 10.1371/journal.pone.0188917 (PMC5737974; doi:10.1371/journal.pone.0188917)
Supplement: S1 Table — (DOCX) [file pone.0188917.s003.docx]

| **Supplementary Table S1. *Sco* mutant FSCs divide faster and persist longer than control FSCs** | | | | | | | | |
| --- | --- | --- | --- | --- | --- | --- | --- | --- |
| **Genotype** | **Total**  **ovarioles** | **Clone Age** | **% of germaria**  **with FC clone^a^** | **Relative** | **% of Partial clone^b^** | **% of Full clone^c^** | **% of Edu^+^ FSC clone^d^** | **% of pH3^+^ FSC**  **clone^e^** |
| **Negative clone** |  |  |  |  |  |  |  |  |
| *FRT40A* control | 186 | 1W | 52.9 ± 5.8 | 100¶ | 46.7 ± 4.0 ∏ | 6.2 ± 2.0 ∏ | 13.2 ± 3.0 | 0 |
|  | 152 | 2W | 40.7 ± 5.3 | 78 | 32.0 ± 5.8 | 8.7 ± 1.3 |  |  |
|  | 205 | 3W | 24.7 ± 3.8*† | 46 | 12.5 ± 3.4*** | 12.3 ± 1.5 |  |  |
| *Sco* | 193 | 1W | 69.3 ± 3.3 | 100 | 59.7 ± 2.9 | 9.5 ± 1.5 | 29.6 ± 3.5ǂ | 0 |
|  | 168 | 2W | 57.6 ± 4.4 | 82 | 38.7 ± 2.7*** | 19.0 ± 4.8 |  |  |
|  | 163 | 3W | 57.8 ± 2.5 | 83 | 25.6 ± 6.8* | 32.2 ± 5.3*ǂ |  |  |

Flies were cultured on a standard food with dry yeast at 25 degrees Celsius. Food was changed daily until dissection.

Clone age: weeks (W) or Days (D) after clone induction.

^a^ Germaria carrying follicle cell (FC) clones, ^b^ germaria carrying a mix of follicle cell clones and wild-type follicle cells, ^c^ germaria carrying only follicle cell clones.

^d^ The percentage of Edu^+^ FSC clones in total FSC clones at the indicated times after clone induction .

^e^ The percentage of pH3^+^ FSC clones in total FSC clones at the indicated times after clone induction.

¶ Percentage of germaria carrying FC clones at the initial time point (1W) remaining at 2W and 3W. The percentage of germaria with FC clones (a) with ∏ Partial clones (b) and full clones (c) at the indicated time points are shown.

*Significant difference as compared to the initial time point: *, *P*<0.05,**, *P*<0.01, ***, *P*<0.001.

ǂ, † Significant difference compared to control at the same time point ǂ, *P*<0.05; †, *P*<0.001

Experiments were analyzed by Student’s *t*-test.
